# The American Dentist in Europe

**Published:** 1895-11

**Authors:** 


THE American Dentist in Europe must not become negligent, or careless if he wished to maintain in security the high position his predecessors made for "American Dentistry." The following was clipped from "*The Swiss and Nice Times*," of Aug. 11th, 1895, and it is only one of many straws which show the direction of the wind of public opinion—Eternal vigilance is the price a reputation for superior skill even among American Dentistry.

It is remarkable what rapid progress the Swiss have made in scientific dentistry of late years. This however is not surprising when we consider the fact of their fine dental colleges, which are on a par with the other colleges and universities of Switzerland. It has been a custom with Swiss students to go to the United States, and there they have learnt all that is taught of interest at the American colleges. Swiss practitioners have superseded the Americans in such cities as Geneva, Zurich and Lucerne. This is easy to understand inasmuch as the Swiss dentists are established and not nomad; they have properly fitted ateliers for mechanical work, an important item in modern dentistry. American dentists, who formerly made large fortunes in this country, are gradually making room for the native, educated dentists. Many of these Americans practise in three different countries in the course of a year, and have little more than a hired room for receiving clients and doing their operations. The practising of talented reputable Swiss dentists has done away with the extortionate charges, which are a special feature of the nomad American.

